# Ectopic expression of Arabidopsis L-type lectin receptor kinase genes *LecRK*-*I.9* and *LecRK*-*IX.1* in *Nicotiana benthamiana* confers *Phytophthora* resistance

**DOI:** 10.1007/s00299-015-1926-2

**Published:** 2016-01-21

**Authors:** Yan Wang, David L. Nsibo, Hagos M. Juhar, Francine Govers, Klaas Bouwmeester

**Affiliations:** Laboratory of Phytopathology, Plant Sciences Group, Wageningen University, Wageningen, The Netherlands; Department of Plant Pathology, Nanjing Agricultural University, Nanjing, People’s Republic of China; Plant–Microbe Interactions, Department of Biology, Faculty of Science, Utrecht University, Utrecht, The Netherlands

**Keywords:** L-type lectin receptor kinases, LecRK, *Phytophthora*, Disease resistance, *Nicotiana* *benthamiana*, Interfamily gene transfer

## Abstract

*****Key message***:**

**Transgenic*Nicotiana benthamiana*lines with constitutive expression of an Arabidopsis lectin receptor kinase gene (*LecRK*-*I.9*or*LecRK*-*IX.1*) show enhanced resistance to*Phytophthora*pathogens, demonstrating conserved gene functionality after interfamily transfer.**

**Abstract:**

In plants, cell surface receptors mediate the first layer of innate immunity against pathogenic microbes. In Arabidopsis several L-type lectin receptor kinases (LecRKs) were previously found to function as *Phytophthora* resistance components. In this study, we determined the functionality of Arabidopsis *LecRK*-*I.9* or *LecRK*-*IX.1* in *Phytophthora* resistance when transferred into the Solanaceous plant *Nicotiana* *benthamiana*. Multiple transgenic lines were generated for each *LecRK* gene and molecular analyses revealed variation in transgene copy number, transgene expression levels and LecRK protein accumulation. Infection assays showed that transgenic *N.* *benthamiana* plants expressing either Arabidopsis *LecRK*-*I.9* or *LecRK*-*IX.1* are more resistant to *Phytophthora* *capsici* and to *Phytophthora* *infestans*. These results demonstrate that Arabidopsis LecRK-I.9 and LecRK-IX.1 retained their *Phytophthora* resistance function when transferred into *N.* *benthamiana*. Therefore, these LecRKs have the potential to function as a complementary *Phytophthora* resistance resource in distantly related plant species next to the canonical *Phytophthora* resistance genes encoding nucleotide-binding leucine-rich repeat proteins.

## Introduction

Plant diseases caused by *Phytophthora* species are widespread and cause enormous economic losses on a large variety of crops (Tyler 2007; Fry 2008; Lamour et al. 2012). Under favorable conditions, *Phytophthora* pathogens reproduce rapidly and become epidemic within a short period of time. *Phytophthora* disease control is costly and often depends on excessive application of preventive fungicides. Hence, development of plant cultivars with durable resistance against different *Phytophthora* species is under high demand. Currently, breeding programs are mainly focused on the exploitation of resistance (*R*) genes that encode intracellular nucleotide-binding leucine-rich repeat (NLR) proteins to restrict *Phytophthora* pathogens (Vleeshouwers et al. 2011). However, these attempts are often hampered by the quick adaptation of *Phytophthora* pathogens that leads to evasion of the *R*-gene mediated recognition (Fry 2008; Vleeshouwers et al. 2011; Rodewald and Trognitz 2013).

Plants also respond to pathogens by activation of plasma membrane-localized receptor-like kinases (RLKs) that function as pattern recognition receptors (PRRs) to initiate defense (Zipfel 2014). Plant resistance mediated by PRRs has been hypothesized to confer broad-spectrum resistance against plant pathogens, but thus far received little attention in resistance breeding of crop plants. One of the largest classes of RLKs comprising potential PRRs are the L-type lectin receptor kinases (LecRKs). LecRKs are ubiquitously present throughout the plant kingdom. Arabidopsis has 45 *LecRK* genes, several of which belong to evolutionary conserved clades whereas others are species- or genus-specific (Bouwmeester and Govers 2009; Hofberger et al. 2015; Wang et al. 2015a).

Thus far, several LecRKs have been found to play a role in plant resistance to different *Phytophthora* pathogens. Arabidopsis LecRK-I.9 was the first one described to function as a *Phytophthora* resistance component (Bouwmeester et al. 2011). To unravel the function of other Arabidopsis LecRKs, a large set of T-DNA insertion mutants covering 36 of the 45 LecRKs was analysed (Wang et al. 2014) and infection assays revealed that mutants of 13 *LecRK*s showed compromised *Phytophthora* resistance. These included mutants of the previously identified LecRK-I.9 and of the two members of clade IX, namely LecRK-IX.1 and LecRK-IX-2. More recently, the latter two were analysed in more detail and this confirmed that they both function as *Phytophthora* resistance component in Arabidopsis (Wang et al. 2015b).

Engineering plants via interfamily transfer of resistance components has the potential to improve disease resistance in crop plants. A successful example is the transfer of Arabidopsis EFR into Solanaceous plants (Lacombe et al. 2010). EFR is a receptor of bacterial elongation factor EF-TU and is restricted to the Brassicaceae family (Kunze et al. 2004; Zipfel et al. 2006). *Nicotiana* *benthamiana* and tomato transgenic plants expressing EFR gained the capacity to respond to EF-Tu and showed enhanced resistance to various bacterial pathogens (Lacombe et al. 2010). In a similar way, Arabidopsis *LecRK-I.9* as transgene in potato was shown to confer enhanced resistance to *Phytophthora infestans* (Bouwmeester et al. 2014). Consistently, transient expression of *LecRK*-*I.9* in *N.* *benthamiana* also resulted in increased resistance to *Phytophthora* pathogens (Bouwmeester et al. 2014) and the same holds for *LecRK-IX.1* and *LecRK-IX.2* (Wang et al. 2015b). Likewise, Arabidopsis *LecRK*-*VI.2* maintained its function in bacterial resistance when expressed in *N.* *benthamiana* (Huang et al. 2014).

The objective of this work was to check whether the Arabidopsis lectin receptor kinase genes *LecRK*-*I.9* and *LecRK*-*IX.1* maintain their functionality in *Phytophthora* resistance when stably integrated as transgene in the distantly related species *N.* *benthamiana*. To this end, transgenic *N.* *benthamiana* plants ectopically expressing either Arabidopsis *LecRK*-*I.9* or *LecRK*-*IX.1* were generated using *Agrobacterium*-mediated transformation. The obtained transgenic lines were subjected to molecular analyses to determine transgene copy number, transgene expression level and LecRK protein accumulation. Thereafter, we monitored the phenotypic changes of these transgenic *N.* *benthamiana* lines in growth and response to different *Phytophthora* pathogens. Since *N. benthamiana* is a model plant amenable for virus-induced gene silencing and is widely used for studying plant-pathogen interactions and protein–protein interactions, these transgenic plants are valuable as experimental tool for further functional analysis of LecRKs.

## Materials and methods

### Plant growth conditions and infection assays

*Nicotiana* *benthamiana* seeds were surface-sterilized by 70 % ethanol and 1 % NaClO, and grown on MS medium (4.4 g MS salt, 20 g sucrose and 8 g agar) or MS medium supplemented with antibiotics in a conditioned growth chamber at 19–21 °C, with a 16 h photoperiod and a relative humidity of 75–80 %. Plants grown in soil were kept in a greenhouse with similar settings. Supplementary light (100 W m^−2^) was applied when the light intensity dropped below 150 W m^−2^.

*Phytophthora* *capsici* isolates LT263 and LT3239 were maintained in the dark on V8 plates at 25 °C (Wang et al. 2013), and *P.* *infestans* isolate 14-3-GFP on rye sucrose agar at 18 °C (Bouwmeester et al. 2014). *P.* *infestans* zoospores were obtained by treating sporulating mycelia with cold water for 3–4 h. For detached-leaf assays, leaves from 5-week-old *N.* *benthamiana* plants were collected and the abaxial sides were inoculated with *P.* *capsici* mycelial plugs (*Ø* 0.5 cm) or 10 µL droplets of a *P.* *infestans* zoospore suspension with a concentration of 5 × 10^5^ zoospores mL^−1^. Inoculated leaves were kept in transparent plastic boxes with high humidity in the dark overnight and thereafter exposed to a condition with a 12 h photoperiod and appropriate temperature settings. Disease severity was monitored by measuring lesion sizes (Vleeshouwers et al. 1999) 3 and 6 days after inoculation with *P.* *capsici* and *P.* *infestans*, respectively.

### *Agrobacterium*-mediated transformation of *N. benthamiana*

*A. tumefaciens* GV3101 carrying the binary vectors pBIN-KS-35S::AtLecRK-I.9-eGFP, pBIN-KS-35S::AtLecRK-IX.1-eGFP (Wang et al. 2015b) and pBIN61-35S::GFP (Fig. 2a) were grown overnight at 28 °C in Yeast Extract Broth with appropriate antibiotics. *A. tumefaciens* cells were pelleted, resuspended and incubated in MMA induction medium (10 mM MES, 10 mM MgCl_2_, 50 μM acetosyringone, pH 5.6) for 3 h. *A.* *tumefaciens* cells were collected by centrifugation and resuspended in MS broth supplemented with 150 μM acetosyringone. Leaf pieces (2–3 cm^2^) were cut from 5-week-old *N. benthamiana* leaves and incubated with *A. tumefaciens* cells for 30 min. Thereafter, leaf discs were dried on filter paper to remove excess *A. tumefaciens* and incubated on regeneration medium consisting of MS salt, 1 mg/L cytokinins 6-BAP (6-benzyl amino purine), 0.1 mg/L auxin NAA (1-naphthaleneacetic acid) and 0.8 % agar for 2 days at 19–21 °C. Leaf pieces were transferred every week to fresh regeneration medium supplemented with 400 mg/L cefotaxime, 200 mg/L vancomycin and 200 mg/L kanamycin until the appearance of plantlets. The generated plantlets were transferred to MS medium containing 200 mg/L kanamycin to allow root development. Upon root generation, plantlets were transferred into soil and kept in the greenhouse for seed harvesting.

### Transgene detection in transgenic *N.benthamiana*

Genomic DNA was isolated using CTAB buffer (0.02 % CTAB, 100 mM Tris–HCl pH 8.0, 20 mM EDTA pH 8.0, 1.4 M NaCl and 1 % PVP) followed by precipitation with isopropanol. RNA was removed by RNaseA. Transgene presence was checked by PCR using specific primers for *AtLecRK*-*I.9* and *AtLecRK*-*IX.1* and a primer matching a flanking sequence in the binary vector (Table 1; Fig. 2a).

**Table 1:** Primers used in this study

Primer		Sequence 5′–3′	Used to/for
NPTII-RT-F	GGAGAGGCTATTCGGCTATG		Check presence of *NPTII*
NPTII-RT-R	TCGTCCTGCAGTTCATTCAG		Check presence of *NPTII*
Nbactin-F	TATGGAAACATTGTGCTCAGTGG		Endogenous control
Nbactin-R	CCAGATTCGTCATACTCTGCC		Endogenous control
Oligo-dT	GACTCGAGTCGACATCGATTTTTTTTTTTTTTT		cDNA synthesis
pGRAB-F1	CCCACTATCCTTCGCAAGACCCTTCC		Check presence of T-DNA
IX.1-RT-F	CAAGGCGAGTAATGTGATGCT		Check presence of *AtLecRK*-*IX.1*; qRT-PCR
IX.1-RT-R	TAACCAAATGTTCCTGCTAACC		qRT-PCR
IX.1-F	TCAAGCCTGGAGCTAATAG		Check presence of *AtLecRK*-*IX.1*
IX.1-R	ACGACCATGTTGAGCACTTG		Check presence of *AtLecRK*-*IX.1*
I.9-RT-F	TTTGCCAGATTTCTCACCATACAC		qRT-PCR
I.9-RT-R	TCTGTTGACTGCCAAGCGTAAG		qRT-PCR
I.9-F	ATGGCTCGTTGGTTGCTTCAG		Check presence of *AtLecRK*-*I.9*
I.9-R	GCTTTGACATCTCGGTGCAGAAC		Check presence of *AtLecRK*-*I.9*

Transgene copy number in T_0_ transgenic lines was determined according to Honda et al. (2002). Briefly, qPCR was performed using genomic DNA as template with specific primers for *AtLecRK*-*I.9*, *AtLecRK*-*IX.1*, the neomycin phosphotransferase II gene (*NPTII*) or *NbActin* (Table 1). The copy number was calculated by normalizing the amplification of *AtLecRK*-*I.9*, *AtLecRK*-*IX.1* or *NPTII* to *NbActin.*

### RNA isolation and qRT-PCR

Total RNA was isolated from leaves collected from 6-week-old T_0_ transgenic *N.* *benthamiana* plants with a NucleoSpin RNA plant Kit (Macherey–Nagel) and thereafter used as template for cDNA synthesis with an oligo-dT primer and a M-MLV reverse transcriptase kit (Promega). qRT-PCR was performed as previously described (Wang et al. 2014). Transcript levels of *AtLecRK*-*I.9* and *AtLecRK*-*IX.1* were normalized to *NbActin*.

### Protein extraction, immunoprecipitation and western blotting

Leaves collected from 6-week-old T_0_ transgenic *N.* *benthamiana* plants were ground in liquid nitrogen and subsequently incubated for 30 min in an extraction buffer containing 150 mM NaCl, 50 mM Tris–HCl pH 8.0, 1.0 % IGEPAL^®^ CA-630 (Sigma) and one protease inhibitor cocktail tablet per 50 mL (Roche). The homogenate was centrifuged at 18,000 rpm for 20 min and the supernatant was incubated with GFP-trap_A^®^ beads (Chromotek) at 4 °C for 1–2 h. After incubation, GFP-beads were spinned down and washed six times with extraction buffer. Proteins were eluted from GFP-trap_A^®^ beads by boiling for 5 min, separated on an 8 % SDS-PAGE gel and electroblotted onto PVDF membrane (Bio-Rad). Accumulation of eGFP-tagged LecRK-I.9 and LecRK-IX.1 was detected by immunoblotting using anti-GFP-HRP (Miltenyi Biotec). Supersignal West Femto Chemiluminescent Substrate (Thermo Scientific) was used for signal development. Coomassie brilliant blue staining was used to indicate the amount of loading.

## Results and discussion

### Generation of transgenic *N.* *benthamiana* ectopically expressing Arabidopsis *LecRK*-*I.9* or *LecRK*-*IX.1*

**Fig. 1:**
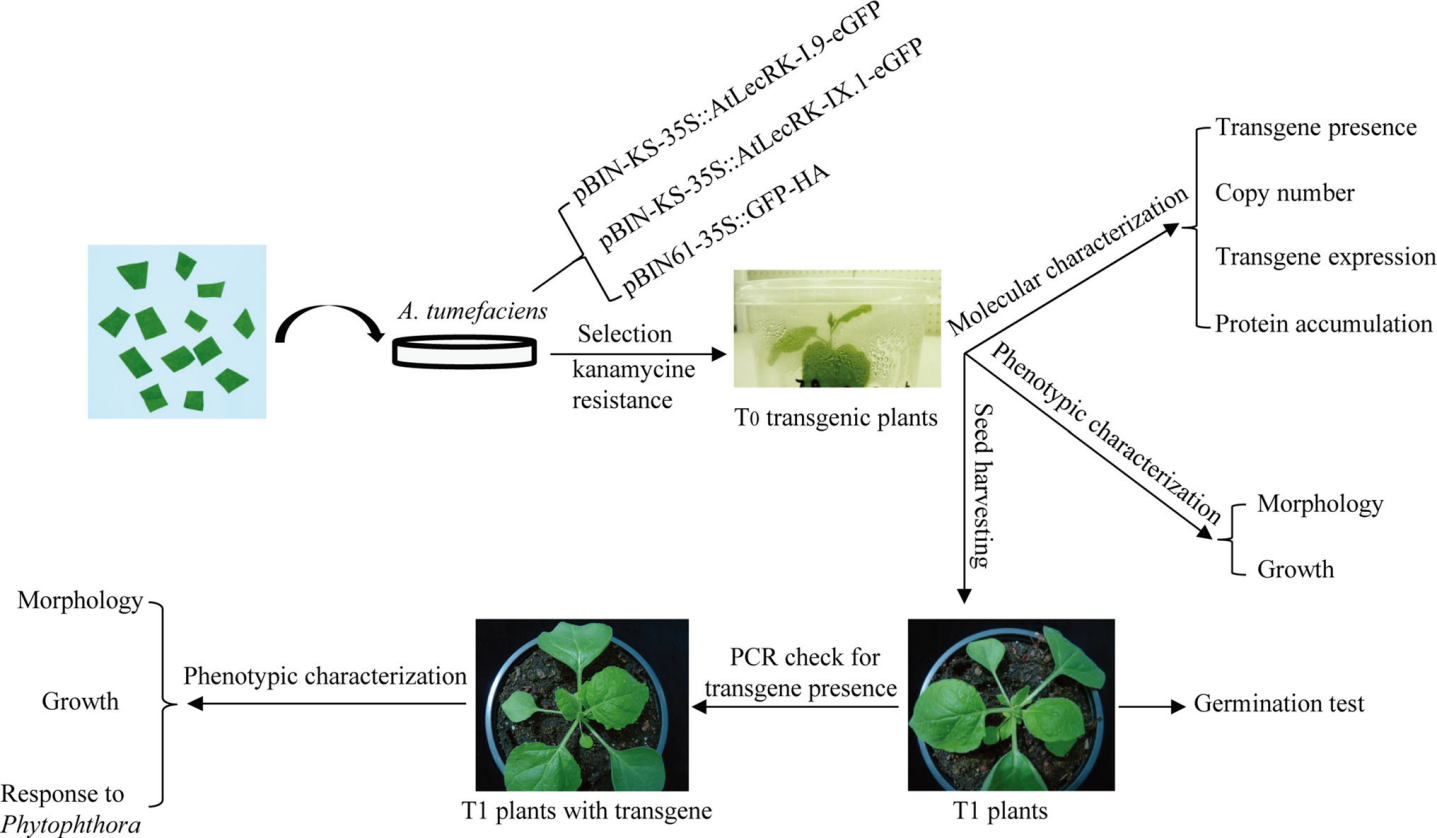
Flowchart of the generation, selection and characterization of transgenic *N.* *benthamiana* lines harboring Arabidopsis *LecRK*-*I.9* or *LecRK*-*IX.1*

**Fig. 2:**
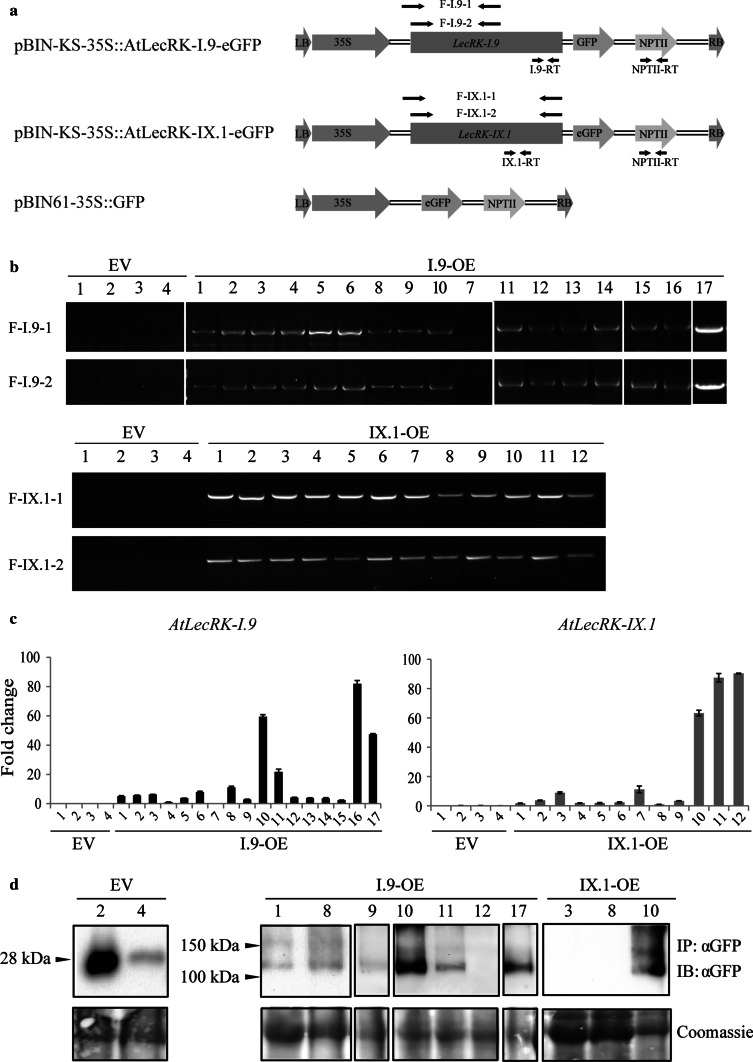
Molecular characterization of transgenic *N.* *benthamiana* lines. **a** Schematic representation of T-DNA regions of the vectors used for *N.* *benthamiana* transformation. PCR amplified fragments and position of the primers are indicated by the *head-to-head arrow pairs*. The fragments *F*-*I.9*-*1*, *F*-*I.9*-*2*, *F*-*IX.1*-*1*, *F*-*IX.1*-*2* and *NPTII*-*RT* were amplified to determine transgene presence in transgenic lines, while fragments *I.9*-*RT* and *IX.1*-*RT* were amplified to monitor transgene mRNA levels. **b** Presence of *AtLecRK*-*I.9* or *AtLecRK*-*IX.1* in transgenic *N.* *benthamiana* lines. Genomic DNA from each line was used as template for amplification with primers indicated in (**a**). **c** Relative quantification of transgene expression levels in transgenic *N.* *benthamiana* lines. Transcript levels were normalized to *NbActin* and values are expressed as mean fold changes (±SD) relative to the transcript level of *AtLecRK*-*I.9* in I.9-OE-4 or the transcript level of *AtLecRK*-*IX.1* in IX.1-OE-8 that was arbitrarily set as 1. **d** GFP, LecRK-I.9-eGFP and LecRK-IX.1-eGFP accumulation in transgenic *N.* *benthamiana* lines. Proteins were immunodetected using anti-GFP-HRP. Coomassie staining was used to indicate the amount of loading in each lane

*Agrobacterium*-mediated transformation of *N.* *benthamiana* with the binary vectors pBIN-KS-35S::AtLecRK-I.9-eGFP, pBIN-KS-35S::AtLecRK-IX.1-eGFP and pBIN61-35S::GFP (Fig. 2a) resulted in 17, 12 and 4 T_0_ kanamycin-resistant lines, respectively. These were named as I.9-OE-1–17, IX.1-OE-1–12 and EV-1–4 (Table 2). The flowchart in Fig. 1 gives an overview of the various steps in the process of selection and analysis of the T_0_ and T_1_ transgenic lines. For the molecular characterization, we first determined whether *AtLecRK*-*I.9* and *AtLecRK*-*IX.1* were successfully transferred into *N.* *benthamiana* by PCR using gene-specific primers (Fig. 2a). Of the selected kanamycin-resistant plants, the four empty vector transgenic lines (EV-1 to EV-4) and one LecRK-I.9 line, i.e. I.9-OE-7, gave no PCR product (Fig. 2b).

**Table 2:** Transgenic *N.* *benthamiana* lines vary in transgene copy number, gene expression and growth

Line no.	Transgene copy number		Relative transgene expression^a^		Morphology (T_0_ transformants vs. wild type)^b^	Germination rate^c^
	*AtLecRK*-*I.9/IX.1*	*NPTII*	*AtLecRK*-*I.9*	*AtLecRK*-*IX.1*		
*EV−*						
1	0	1	n.d.	n.d.	–	100
2	0	2	n.d.	n.d.	–^e^	100
3	0	5	n.d.	n.d.	–	100
4	0	2	n.d.	n.d.	–^e^	100
I.9-OE−						
1	1	1	5.0		Slightly smaller^e^	32
2	2	2	5.6		Smaller plants with curly leaves	60
3	3	3	6.2		Smaller plants with compacted rosette^e^	40
4	3	3	1.0		–	60
5	1	1	3.7		–^e^	100
6	4	4	7.8		–	55
7	0	1	n.d.		–^e^	/^d^
8	1	1	11.1		–	60
9	1	1	2.8		Smaller^e^	85
10	5	6	59.4		Smaller^e^	95
11	4	6	21.7		Smaller^e^	65
12	1	1	4.1		Smaller plants with compacted rosette, thick leaves^e^	55
13	2	2	3.9		–	50
14	2	2	3.6		–	70
15	1	1	2.3		Smaller^e^	60
16	1	1	81.9		Smaller plants with compacted rosette	35
17	3	3	47.3		Slightly smaller and curly leaves	85
*IX.1-OE−*						
1	2	2		1.7	–^e^	100
2	1	2		3.7	Smaller plants with curly round leaves^e^	100
3	1	2		9.0	Smaller^e^	100
4	2	2		2.0	–^e^	100
5	2	3		2.0	–^e^	100
6	1	2		2.4	–	100
7	1	1		11.2	Smaller^e^	100
8	2	2		1.0	–	100
9	1	1		3.4	–	100
10	1	1		63.2	Smaller plants with old leaves showing necrosis^e^	100
11	1	1		87.3	Smaller plants with old leaves showing necrosis	100
12	1	1		90.3	Smaller plants with old leaves showing necrosis^e^	100

^a^ *n.d*. not detectable

^b^ –, no difference compared with wild-type *N. benthamiana*

^c^Percentage of germinated seeds of T_1_ progeny lines (*n* = 18–24) after 3 days on MS

^d^ / not tested

^e^Similar morphology in T_1_ progeny lines harboring the transgene

To determine transgene copy number, we performed qPCR analysis. For both *AtLecRK*-*I.9* and *AtLecRK*-*IX.1*, the copy number in transgenic lines ranged from 0 to 5 and this number was not always consistent with that of the determined *NPTII* copy number (Table 2). In the transgenic lines I.9-OE-7, I.9-OE-10, I.9-OE-11, IX.1-OE-2, IX.1-OE-3, IX.1-OE-5 and IX.1-OE-6, more copies were detected for *NPTII* than for *LecRK*s, indicating the presence of truncated T-DNA fragments. This likely explains why the T_0_ line I.9-OE-7 lacks *AtLecRK*-*I.9* but is still kanamycin-resistant.

Expression of *AtLecRK*-*I.9* and *AtLecRK*-*IX.1* in the T_0_ plants was determined by quantifying mRNA levels using qRT-PCR. For both *AtLecRK*-*I.9* and *AtLecRK*-*IX.1*, the expression level varied among individual transgenic lines (Fig. 2c). Variations in transgene expression level in stable transgenic lines have often been attributed to the site(s) of transgene insertion and transgene copy number (Kole et al. 2010). In T_0_ plants, however, no correlation was found between copy number and transgene expression level. Transgene expression level varied among individual transgenic lines with the same transgene copy number. Some of the transgenic lines with a single copy of the transgene showed even higher expression levels than those with two or more copies. For example, line I.9-OE-16 contains a single *AtLecRK*-*I.9* copy but showed the highest transgene expression level of all I.9-OE lines. Even transgenic lines with the same transgene number, e.g. line IX.1-OE-3 and IX.1-OE-11, showed quite different expression levels (Fig. 2c). To determine whether the transgenic lines produce the LecRK proteins, we isolated proteins and performed western blot analysis using GFP antibody. For this analysis we selected a subset of seven I.9-OE lines and three IX.1-OE lines that varied in transgene copy number and expression. In two EV lines (i.e. EV-2 and EV-4), different amounts of GFP with the expected size around 28 kDa were detected. In the I.9-OE lines, variable amounts of LecRK-I.9-eGFP were detected (Fig. 2d) and comparison with the expression levels (Fig. 2c) suggests that the accumulation of the LecRK-I.9 is correlated with the transgene expression level. LecRK-IX.1-eGFP was only detected in one of the three IX.1-OE lines, and this is the one, IX.1-OE-10, with the highest transgene expression level of the three selected IX.1-OE lines (Fig. 2d).

### Morphology and growth alterations of I.9-OE and IX.1-OE lines

**Fig. 3:**
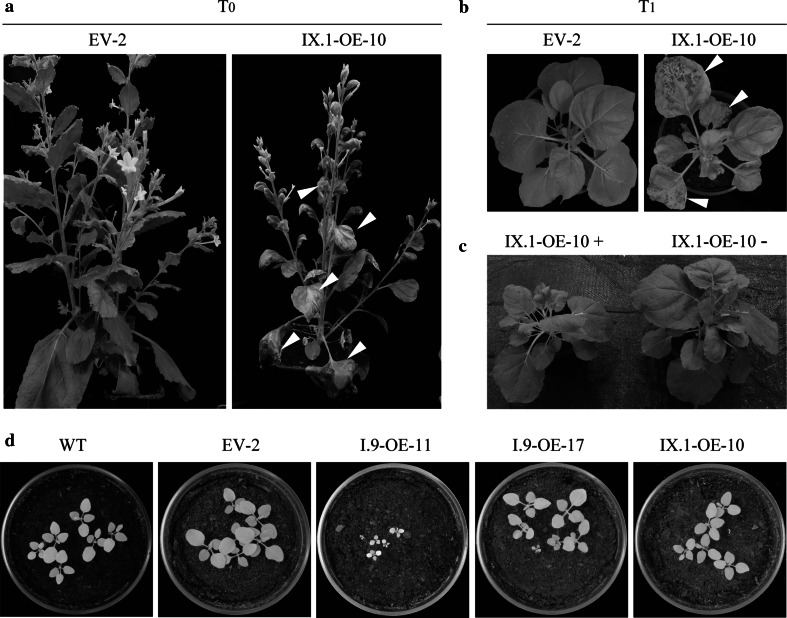
Morphology of transgenic *N.* *benthamiana* lines. **a** The T_0_ *N.* *benthamiana* IX.1-OE-10 plant, but not the T_0_ EV-2 plant displayed cell death. Ten-week-old plants were photographed. **b** The T_1_ *N.* *benthamiana* IX.1-OE-10 plant, but not the EV-2 plant developed cell death. Six-week-old plants were photographed. **c** T_1_ progeny of the IX.1-OE-10 line harboring *AtLecRK*-*IX.1* (+) is smaller in size than the T_1_ progeny without *AtLecRK*-*IX.1* (−). Six-week-old plants were photographed. **d** Germination of seeds harvested from untransformed and transgenic *N.* *benthamiana* plants. Six seeds were sown in each pot. Two-week-old seedlings were photographed

In a previous study, it was reported that overexpression of *AtLecRK*-*I.9* in Arabidopsis led to more compact rosettes with smaller and slightly wrinkled leaves (Bouwmeester et al. 2011). In line with this, ectopic expression of *AtLecRK*-*I.9* in potato also led to developmental defects, such as aberrant leaf morphology (Bouwmeester et al. 2014). In this study, the T_0_ transgenic *N. benthamiana* plants were monitored for growth and developmental alterations starting from 1-week after transfer into soil until seed set (Table 2; Fig. 1). All the four EV lines showed normal development; i.e. leaf morphology, branching and plant height were similar to untransformed *N.* *benthamiana*. In contrast, several I.9-OE lines were smaller in size and displayed more compact rosettes or curly leaves. These phenotypic changes, however, were not correlated with *AtLecRK*-*I.9* expression levels, but this could be due to a combined effect of varying transgene copy numbers, transgene insertion sites and transgene expression levels in the different transgenic lines. Three out of the twelve IX.1-OE lines, namely IX.1-OE-10, IX.1-OE-11 and IX.1-OE-12, showed spontaneous cell death in leaves of over 5-week-old plants (Fig. 3a), a phenomenon that was not observed in any of the *N. benthamiana* lines expressing *LecRK*-*I.9*. Considering that these three IX.1-OE lines showed much higher *AtLecRK*-*IX.1* expression levels than the rest, the cell death phenotype might be attributed to the elevated *AtLecRK*-*IX.1* expression. This is supported by our previous observations reported in Wang et al. (2015b) that transient expression of *AtLecRK*-*IX.1* in *N. benthamiana* also enhances cell death. Moreover, a similar cell death phenotype was found when *AtLecRK*-*IX.1* was overexpressed in Arabidopsis, and this was also shown to be correlated with *AtLecRK*-*IX.1* expression levels (Wang et al. 2015b).

To determine whether the observed LecRK-mediated phenotypes are maintained in the offspring, two EV lines, nine I.9-OE lines and eight IX.1-OE lines were propagated and their segregating progeny (T_1_ plants) was assayed for transgene presence and alteration in morphology and development of spontaneous cell death. As indicated in Table 2, the phenotypes in T_1_ plants containing the transgene were similar to those observed in T_0_ lines. Spontaneous cell death was only found in progeny of IX.1-OE lines that has high transgene expression (Fig. 3b; Table 2). These plants also showed retarded growth when compared to those without *AtLecRK*-*IX.1* (Fig. 3c) or with low transgene expression (Table 2). Based on these observations, we conclude that the observed morphological and cell death phenotypes in these lines are due to the presence of the *AtLecRK*-*IX.1* transgene and not to a random gene insertion effect. In a previous study, we showed that mutation of the catalytic RD-motif within LecRK-IX.1 abolishes induction of cell death and pathogen resistance (Wang et al. 2015b). Hence, we anticipate that the induced cell death is either directly mediated by LecRK-IX or indirectly through constitutive activation of defense.

For all the EV and IX.1-OE lines, seeds harvested from T_0_ plants have a similar germination efficiency as untransformed *N.* *benthamiana* when grown in soil or on MS medium (Table 2; Fig. 3d). However, 10 out of the 16 I.9-OE lines showed severe defects in seed germination, with a germination rate of 60 % or lower (Table 2). Also here, the severity of the phenotype does not correlate with *AtLecRK*-*I.9* expression levels in the various transgenic lines.

### I.9-OE and IX.1-OE lines show enhanced resistance to *Phytophthora* pathogens

**Fig. 4:**
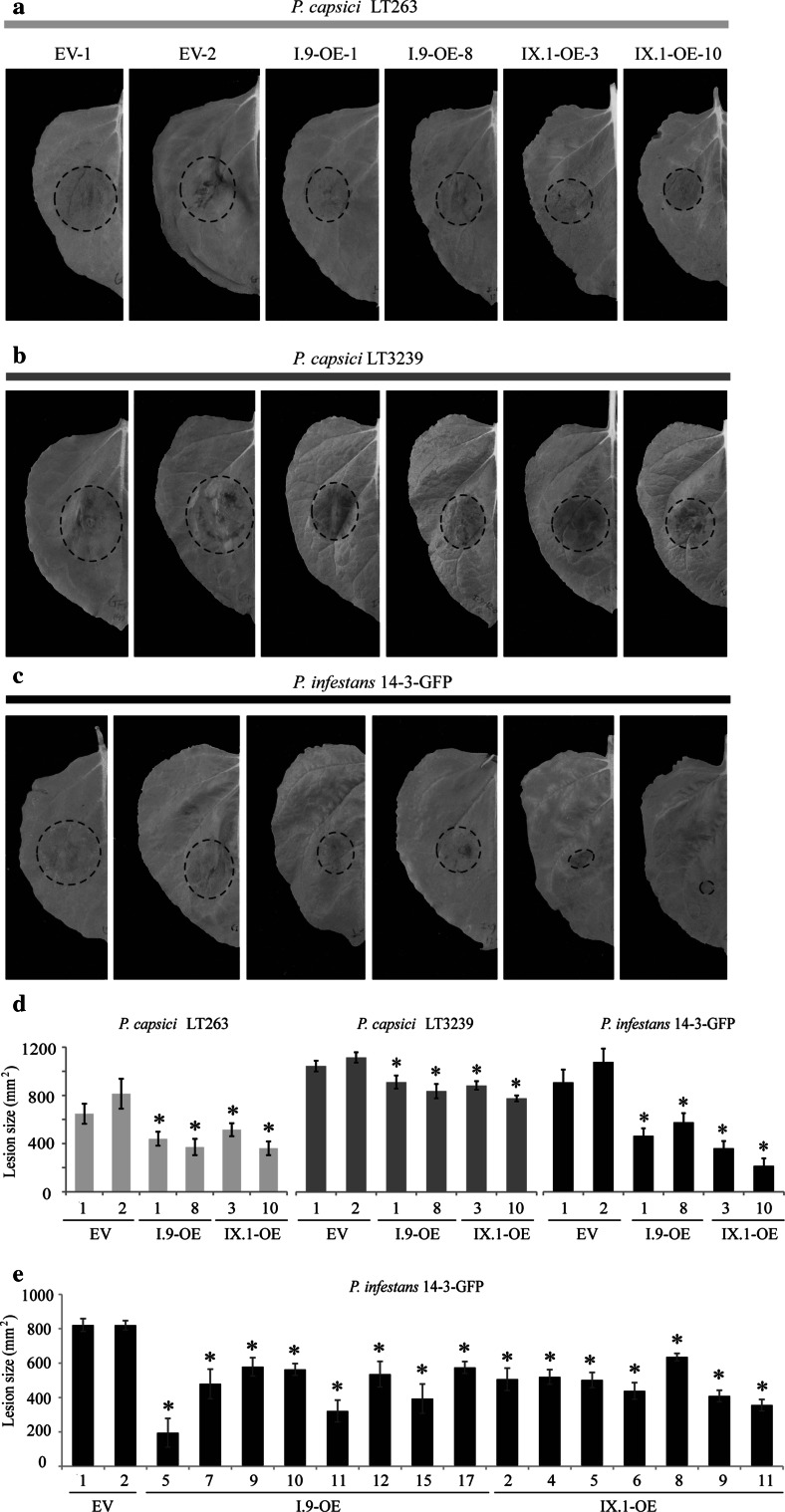
Infection assays on transgenic *N.* *benthamiana* lines with different *Phytophthora* pathogens. **a**–**c** Disease symptoms on transgenic *N.* *benthamiana* EV, I.9-OE and IX.1-OE lines 3 days after plug-inoculation with *P.* *capsici* LT263 (**a**) or LT3239 (**b**), or 6 days after zoospore-inoculation with *P.* *infestans* 14-3-GFP (**c**). Lesions are indicated by *black circles*. **d** Average lesion sizes on *N.* *benthamiana* plants upon inoculation with *Phytophthora* pathogens. Each experiment included 12–20 inoculation sites. *Bars* represent the mean lesion sizes (±SE). *Asterisks* indicate significant difference in lesion sizes (*p* < 0.01) compared to the EV lines based on One-way ANOVA with Tukey’s HSD test. Infection assays were repeated three times with both *P.* *capsici* isolates and twice with *P.* *infestans* with similar results. **e** Average lesion sizes on *N.* *benthamiana* plants inoculated with *P. infestans*. Each experiment included 12–20 inoculation sites. *Bars* represent the mean lesion sizes (±SE). *Asterisks* indicate significant difference in lesion sizes (*p* < 0.01) compared to the EV lines based on One-way ANOVA with Tukey’s HSD test. Infection assays were repeated twice with similar results

Both LecRK-I.9 and LecRK-IX.1 were previously shown to function in *Phytophthora* resistance in Arabidopsis (Bouwmeester et al. 2011; Wang et al. 2015b). In order to determine whether both LecRKs retain their function in *Phytophthora* resistance, we checked the response of *N.* *benthamiana* I.9-OE and IX.1-OE lines upon inoculation with *P.* *capsici* and *P.* *infestans*. Leaves from T_1_ progenies harboring the transgenes were used for infection assays. It has to be noted that for lines with a cell death phenotype only leaves without any visible cell death symptoms were used for infection assays. Upon plug-inoculation with *P.* *capsici* LT263 or LT3239, smaller lesions were found on the I.9-OE and IX.1-OE lines when compared to EV-1 and EV-2 (Fig. 4a, b), indicating that constitutive expression of *AtLecRK*-*I.9* or *AtLecRK*-*IX.1* in *N.* *benthamiana* enhances resistance to different isolates of *P.* *capsici*. This increased resistance was also found in I.9-OE and IX.1-OE lines when inoculated with *P.* *infestans* (Fig. 4c, d). As shown in Fig. 4e, all tested transgenic lines showed reduced lesion sizes when compared with those on EV control plants. There was however, no indication for a correlation between lesion size and the level of transgene expression (Fig. 2; Table 2). For example, lesion sizes on lines with high transgene expression levels (e.g. I.9-OE-10 and I.9-OE-17) were found to be comparable with those displayed on lines that have low transgene expression levels (e.g. I.9-OE-9). On the other hand lines that have one transgene copy with similar transgene expression levels were found to vary significantly in lesions sizes (e.g. I.9-OE-5 and I.9-OE-7). These findings show that the resistance phenotype in these transgenic lines is regulated at different levels and cannot be entirely attributed to the level of transgene expression or the transgene copy number.

## Conclusions

In this study, multiple transgenic *N.* *benthamiana* lines with constitutive expression of Arabidopsis *LecRK*-*I.9* or *LecRK*-*IX.1* were obtained. Transgenic lines varied in transgene copy number, transgene expression level and protein accumulation. Ectopic expression of either *AtLecRK*-*I.9* or *AtLecRK*-*IX.1* in *N.* *benthamiana* increased the resistance to different *Phytophthora* species. Our results suggest that Arabidopsis *LecRK*-*I.9* and *LecRK*-*IX.1* maintained their function in *Phytophthora* resistance when transferred into *N.* *benthamiana*, which is in line with results that we obtained previously with transgenic potato plants expressing *LecRK*-*I.9* (Bouwmeester et al. 2014). These findings suggest that *LecRKs* could be used as complementary resistance components, in combination with canonical NLR-encoding *R* genes, for engineering broad-spectrum disease resistance to *Phytophthora* pathogens. However, ectopic expression of both *LecRK*s also caused several adverse effects on plant fitness, such as curly leaves, leaf necrosis or reduced plant size. In the case of *LecRK*-*IX.1*, its function in *Phytophthora* disease resistance is independent of that in plant cell death induction (Wang et al. 2015b). Therefore, we anticipate that it should be possible to optimize the receptors in such a way that downstream signaling does no longer cause plant growth alterations while the LecRK-mediated disease resistance is maintained. The transgenic *N.* *benthamiana* lines that we describe in this study can be used as a valuable experimental tool for further analysis of the components required for LecRK-mediated resistance and plant growth alterations, for example via the virus-induced gene silencing or protein complex pull-down assays.

### **Author contribution statement**

Y.W., F.G. and K.B. conceived and designed research. Y.W., D.L.N. and H.M.J. carried out the experiments and analysed data. Y.W., F.G. and K.B. wrote the manuscript. All authors read and approved the manuscript.
